# Genomic-transcriptomic analysis identifies the Syrian hamster as a superior animal model for human diseases

**DOI:** 10.1186/s12864-025-11393-4

**Published:** 2025-03-24

**Authors:** Chuchu Wang, Zhenguo Cheng, Jinxin Miao, Xia Xue, Yunshu Dong, Li Zhao, Haoran Guo, Jianyao Wang, Zhizhong Wang, Shuangshuang Lu, Guangming Fang, Ying Peng, Yafei Zhai, Zhongxian Zhang, Dongling Gao, Zhimin Wang, Pengju Wang, Lirong Zhang, Louisa S Chard Dunmall, Jun Wang, Wenxue Tang, Xiaowei Li, Zhongren Ding, Xiaoyan Zhao, Ling Li, Nicholas R. Lemoine, Zhongde Wang, Daniel Tonge, Wenjie Tan, Jianzeng Dong, Yaohe Wang

**Affiliations:** 1 School of Life Sciences, Zhengzhou University, Zhengzhou, 450001 People’s Republic of China; 2 Sino-British Research Centre for Molecular Oncology, National Centre for International Research in Cell and Gene Therapy, Academy of Medical Sciences, Zhengzhou University, Zhengzhou, 450052 People’s Republic of China; 3 Academy of Chinese Medicine Science, Henan University of Chinese Medicine, Zhengzhou, 450000 People’s Republic of China; 4 Henan Key Laboratory for Helicobacter Pylori and Digestive Tract Microecology, The Fifth Affiliated Hospital of Zhengzhou University; Institute of Rehabilitation Medicine, Henan Academy of Innovations in Medical Science; Tianjian Laboratory of Advanced Biomedical Sciences, Zhengzhou University, Zhengzhou, Henan 450001 China; 5 Department of Obstetrics and Gynecology, Peking University Third Hospital, Beijing, China; 6 National Institute for Viral Disease Control and Prevention, China CDC, Beijing, 102206 People’s Republic of China; 7 Department of Cardiology, Centre for Cardiovascular Diseases, Henan Key Laboratory of Hereditary Cardiovascular Diseases, The First Affiliated Hospital of Zhengzhou University, Zhengzhou University, Zhengzhou, 450052 People’s Republic of China; 8 School of Basic Medical Sciences, Academy of Medical Sciences, Zhengzhou University, Zhengzhou, 450001 People’s Republic of China; 9 Centre for Cancer Biomarkers & Biotherapeutics, Barts Cancer Institute, Queen Mary University of London, London, EC1M 6BQ UK; 10 Centre for Precision Medicine, Academy of Medical Sciences, Zhengzhou University, Zhengzhou, 450052 People’s Republic of China; 11 Department of Animal, Dairy and Veterinary Sciences, College of Agriculture and Applied Sciences, Utah State University, Logan, UT USA; 12 School of Life Sciences, Keele University, Keele, Staffordshire ST5 5BG UK; 13 Department of of Cardiology, Beijing Anzhen Hospital, Capital Medical University, No. 2, Anzhen Road, Chao Yang District, Beijing, 100029 People’s Republic of China

**Keywords:** Syrian hamster, Omics, SARS-COV-2, Cardiovascular disease, Cancer, Model animal

## Abstract

**Background:**

The Syrian hamster (*Mesocricetus auratus*) has shown promise as a human diseases model, recapitulating features of different human diseases including COVID-19. However, the landscape of its genome and transcriptome has not been systematically dissected, restricting its potential applications.

**Results:**

Here we provide a complete analysis of the genome and transcriptome of the Syrian hamster and found that its lineage diverged from that of the Chinese hamster (*Cricetulus griseus*) around 29.4 million years ago. 21,387 protein-coding genes were identified, with 90.03% of the 2.56G base pair sequence being anchored to 22 chromosomes. Further comparison of the transcriptomes from 15 tissues of the Syrian hamster revealed that the Syrian hamster shares a pattern of alternative splicing modes more similar to humans, compared to rats and mice. An integrated genomic-transcriptomic analysis revealed that the Syrian hamster also has genetic and biological advantages as a superior animal model for cardiovascular diseases. Strikingly, several genes involved in SARS-COV-2 infection, including *ACE2,* present a higher homology with humans compared to other rodents and show the same function as their human counterparts.

**Conclusion:**

The detailed molecular characterisation of the Syrian hamster in the present study opens a wealth of fundamental resources from this small rodent for future research into human disease pathology and treatment.

**Supplementary Information:**

The online version contains supplementary material available at 10.1186/s12864-025-11393-4.

## Background

Animal models have made a vital contribution to the advancement of medical knowledge and improvement of human health and provide a fundamental resource for understanding human diseases [1]. These models enable studies of aetiological agents, identification of emerging pathogens, observation of disease pathogenesis, and evaluation of the safety and efficacy of vaccine and therapeutic candidates for treatment of disease [2–4].

The Syrian hamster is an outstanding model for the study of high-risk human infectious diseases due to its high sensitivity to pathogens and the induction of similar immune responses to those seen in humans and non-human primates (NHPs) [3, 5]. The Syrian hamster has been used as an experimental model to mimic infectious diseases including SARS, Ebola virus disease (EBV) and the COVID-19 pandemic [6–9]. It has been found that the pathological features associated with SARS-CoV-2 infection in Syrian hamsters closely resemble those seen in humans, including rapid weight loss and severe lung pathology [7, 9, 10]. Of particular interest is that infection with SARS-CoV-2 in hamsters reflects some of the age demographic differences seen in COVID-19 patients [7, 11]. The Syrian hamster has also been demonstrated as an ideal model for the study of cancer and cardiovascular diseases [3, 12, 13]. The survival rate of severe cardiovascular diseases is limited by the lack of understanding of the precise mechanism of heart failure and its often-sudden incidence, especially in young adults [14, 15]. Thus, a tractable animal model for cardiovascular disease is urgently required to improve therapeutic outcomes. Although the mouse is a common model animal for studying atherosclerosis-related diseases, the natural genetic divergence between mouse and human limits its power in certain scenarios, such as when investigating key metabolic enzyme defects and genetic background differences [16–18]. Compared with mice, the low cost and fast growth rate of Syrian hamsters has advanced its application in experimental studies [19–21]. More notably, the cardiomyopathic Syrian hamster shows a physical response to a high fat/cholesterol diet in a similar manner to humans, with high levels of atherogenic lipoproteins observed under these feeding conditions [22]. The lack of need for genetic engineering to represent human-like advanced lesions and high cholesterol levels also suggests the Syrian hamster as an effective and straightforward model for cardiac hypertrophy and heart failure. The genes associated with immune and cardiovascular symptoms in the Syrian hamster show similar expression patterns to humans, including the natriuretic protein hormones NPPA and NPPB and the genes related to the sarcoplasmic reticulum calcium pump [23].

A lack of detailed molecular characterisation at the genomic and transcriptomic level of the Syrian hamster has become a major obstacle to its use in research, limiting its utility for fully studying human diseases. Although different versions of the assembled genome of the Syrian hamster were previously released (Table S1), they were sequenced from a single female hamster without any Y-linked genes, moreover, the genome assemblies and annotations were based on the scaffold level, which failed to provide a chromosomal landscape for understanding the genetic information of the Syrian hamster [24]. Thus, a comprehensive analysis of the “-omics” of the Syrian hamster will provide a foundation for its use in studying human diseases at the genetic level.

The present study provides a high-quality chromosomal-level assembled genome from a male adult Syrian hamster based on high-throughput sequencing. Besides the comprehensive gene annotation, we performed comparative transcriptomics of 15 organs/tissues from Syrian hamsters and characterized the hamster genes that are involved in SARS-COV-2 infection and human coronary artery disease (CAD) by analysing gene homology, exon skipping, specific organ expression and validation of biological function. Our study indicates that the Syrian hamster is the optimal animal model to recapitulate SARS-COV-2 infection and human cardiovascular diseases and provides a fundamental and resource-rich dataset for using the Syrian hamster as an animal model for disease evaluation.

## Methods

### Animal studies

Syrian hamsters (4–5 wk old, 80 g in weight) were purchased from Beijing Vital River Laboratory Animal Technology Co. (Beijing, China). The animals were maintained under specific pathogen-free, 14 h light/10 h dark cycle (room temperature 23 ± 0.5 °C humidity 40%−60%) conditions and the animals had free access to feed irradiated chow and water. All animal care and experiments were approved by the Ethical Committee of the Zhengzhou University and were in accordance with the Provision and General Recommendation of Chinese Experimental Animals Administration Legislation. The data reported in this manuscript is in accordance with ARRIVE guidelines.

### DNA sampling and sequencing

Genomic DNA was extracted from muscle tissue of a male golden Syrian hamster and was used to prepare an Illumina paired-end library, nanopore library and Hi-C library. For the establishment of the Illumina paired-end library, genomic DNA was fragmented by Covaris, and then end-polished. dAMP(deoxyadenosine monophosphate) and adaptors were added for constructing a sequencing library with an insert size of 350 bp. Paired-end sequencing was performed with the Illumina NovaSeq 6000 platform according to the manufacturer’s instructions and produced a total of 307.53 Gb (98.25 ×) raw data for the survey and correction of the Syrian hamster genome. Quality control was performed on the Illumina raw data to ensure Q30 > = 80% through in-house Perl scripts. Specifically, reads with the adaptors, reads with N bases more than 10%, paired reads with Q < 5 base pairs more than 20% and duplicate reads were removed to obtain clean reads. We obtained a total of 306.92 Gb (~ 98 ×) clean reads for downstream analysis. All clean reads were applied to estimate the hamster genome’s size and heterogeneity based on the k-mer distribution analysis using a k value of 17. For Nanopore libraries, high molecular weight genomic DNA was processed with a Ligation Sequencing Kit following the manufacturer’s protocol including steps: (a) DNA repair and end-preparation; (b) ligation of sequencing adaptors and clean-up; (c) quality control. Nanopore libraries were sequenced on PromethION according to the manufacturer’s instruction.

We constructed a high-throughput chromosome conformation capture (Hi-C) library for the hamster genome. Muscle tissue was fixed with formaldehyde to induce DNA cross-linking. After digestion with a restriction endonuclease, DNA was biotinylated by biotin-14-dCTP and then ligated by T4 DNA Ligase to form chimeric junctions. The ligated DNA was reverse cross-linked and physically sheared into 300–600 bp fragments. The DNA fragments were purified through biotin-streptavidin-mediated pulldown and were blunted-end repaired and A-tailed to construct Hi-C sequencing libraries. The Hi-C libraries were quantified and sequenced on the Illumina NovaSeq 6000 and HiSeq platform (Novogene Biotech Cor., Ltd, Beijing China). The Hi-C libraries generated 336.87 Gb of raw data, and 336.06 Gb of clean data were left after quality control.

### Nanopore reads assembly, correction and validation

A total of 314.37 Gb Nanopore sequencing data was obtained to perform de novo genome assembly by using the long-read genome assembler wtdbg2 (v2.3, parameters: input_fofn = input.fofn, ref = '', data_type = ONT, genome_size = 3.0, max_depth = 60, node-drop = 0.25, node-len = 1536, node-max = 200, brute_force = 1) [25]. The following strategies were performed: (a) alignment algorithms: Kmer-Bin-Mapping; (b) assembling algorithms based on fuzzy-Bruijin graph (FBG); (c) consensus correction was performed using Nanopore data with Racon (v1.3.1) and the following parameters: -u -t 40 [26]. To further decrease the overall error rate, we performed consensus correction alignment using Illumina reads mapped with BWA −0.6 and Plicon (v1.22, parameters: -Xmx300G –diploid –threads 20) [27]. To evaluate uniformity of the sequencing data, we estimated the mapping rate of the Illumina reads and coverage/average sequencing depth of the genome. We employed CEGMA (Core Eukaryotic Genes Mapping Approach: http://korflab.ucdavis.edu/dataseda/cegma/) and BUSCO (Benchmarking Universal Single-Copy Orthologs: http://busco.ezlab.org/) to evaluate the completeness of the genome assembly.

### Chromosome-scale assembly of Syrian hamster genome with Hi-C mapping information

Hi-C high-quality clean data were filtered from Hi-C clean data with the same standard for Illumina raw reads. We used BWA −0.6 to map the clean Hi-C reads to the draft assembled sequence. We used SAMTOOLS (v0.1.18) [28] to select high mapping quality reads that aligned within 500 bp of a restriction site. We excluded reads with low mapping quality, multiple hits or duplication. Lachesis (version-201701) (https://github.com/shendurelab/LACHESIS) was used to cluster contigs into chromosome groups, order contigs within chromosomes and orient the contigs with parameters “RE_SITE_SEQ = GATC, CLUSTER_N = 22, CLUSTER_MIN_RE_SITES = 325”.

### Genome annotation

The repeats in the genome consisted of tandem repeats and interspersed repeats (also known as transposable elements, TEs). Tandem Repeat sequences and transposable elements in the hamster genome were identified using an integrated strategy incorporating de novo and homology-based approaches at the DNA and protein levels. A de novo repeat library for the hamster genome was generated using the combination of three programs RepeatModeler (http://www.repeatmasker.org/RepeatModeler/), RepeatScout (http://www.repeatmasker.org/) and LTR_FINDER (http://tlife.fudan.edu.cn/tlife/ltr_finder/).

TEs were identified by a combination of the de novo repeat library and RepBase library (Bao et al., 2015) by Uclust with 80–80-80 principle and annotated by RepeatMasker. RepeatProteinMask was used to search the TE protein database using WU-BLASTX algorithms in order to identify and classify TEs at the protein level. We integrated the results and generated a consensus and non-redundant TEs library (combined TEs) [7].

### Gene prediction

Structural annotation of gene models was constructed by incorporating de novo, homology-based and RNA-seq-assisted prediction. The de novo prediction was implemented using Augustus(http://bioinf.uni-greifswald.de/augustus/) [29], GlimmerHMM (http://ccb.jhu.edu/software/glimmerhmm/) [30], SNAP(https://github.com/KorfLab/SNAP) [31], Geneid [32] and Genscan (http://argonaute.mit.edu/GENSCAN.html) [33]. For homology-based prediction, protein sequences from seven sequenced vertebrates *Cricetulus griseus* (*Cgr*), *Microtus ochrogaster* (*Moc*), *Rattus norvegicus* (*Rno*), *Mus musculus* (*Mmu*), *Mus caroli* (*Mca*), *Peromyscus maniculatus* (*Pma*) and *Homo sapiens* (*Hsa*), were initially mapped onto the hamster genome using blast(http://blast.ncbi.nlm.nih.gov/Blast.cgi). Homologous genome sequences were then aligned against matched protein using GeneWise (http://www.ebi.ac.uk/~birney/wise2/) to obtain accurate spliced alignments.

To obtain additional support for the structural annotation of gene models, 11 Illlumina RNA libraries from 11 tissues and a PacBio SMRT RNA-seq library of eight mixed tissues of Syrian hamster were sequenced and processed. Raw reads were submitted in Genbank (PRJNA662719, Table S2). Illlumina RNA-seq data were mapped to genome using Tophat (version 2.0.8). Cufflinks (version 2.1.1) (http://cufflinks.cbcb.umd.edu/) was used to assemble transcripts to gene models. PacBio RNA-seq data subreads.bam was processed using css from SMRTlink _6.0.0.47841 with parameters “–skip-polish –num-threads 30”. The PacBio bam file was then clustered and polished using isoseq3_0.7.2 with parameters “–rq-cutoff 0.99, –coverage 60”. Furthermore, the assembled transcripts based on Illlumina RNA-seq data and PacBio SMRT RNA-seq data were used to identify candidate protein-coding regions with the Program to Assemble Spliced Alignments (PASA, https://github.com/PASApipeline/PASApipeline/wiki) [34]. Finally, consensus gene sets were integrated using three respective annotation files with EVidenceModeler (EVM, http://evidencemodeler.github.io/) [34].

### Gene function annotation

The functional annotation of the predicted genes of hamster was performed by alignment to the protein databases using BLASTALL and KAAS. The protein databases involved in the present study were SwissProt (http://www.uniprot.org/), Nr (http://www.ncbi.nlm.nih.gov/protein), Pfam (http://pfam.xfam.org/), KEGG (http://www.genome.jp/kegg/), and InterPro (https://www.ebi.ac.uk/interpro/).

### Non-coding RNA annotation

Putative tRNAs were annotated by tRNAscan-SE (http://lowelab.ucsc.edu/tRNAscan-SE/). miRNAs and snRNAs were predicted using INFERNAL 1.1 (http://infernal.janelia.org/) by searching against the Rfam database (available via the website at http://rfam.sanger.ac.uk and through our mirror at http://rfam.janelia.org). rRNAs were detected by performing blast search with rRNA sequences from closely related species.

### Gene family classification

We analyzed the protein-coding genes from hamster and 15 other vertebrate species, including *Canis lupus familiaris*, *Sus scrofa*, *Cavia porcellus*, *Heterocephalus glaber*, *Rattus norvegicus*, *Mus caroli*, *Mus musculus*, *Peromyscus maniculatus*, *Microtus ochrogaster*, *Cricetulus griseus*, *Spalax galili*, *Homo sapiens*, *Macaca mulatta*, *Macaca fascicularis* and *Loxodonta africana* (Table S3). All data were downloaded from NCBI and Ensembl. Only the longest protein sequence was kept for further analysis if there were alternative splicing isoforms. Genes encoding proteins with less than 30 amino acids were excluded. The identification of orthologous groups was performed with OrthoMCL (Version 2.0, http://orthomcl.org/orthomcl/.) using a Markov Cluster algorithm to group (putative) orthologs and paralogs. We identified a total of 19,615 gene families and 6,620 single-copy orthologues.

### Phylogenetic analysis

Phylogenetic analysis was performed using 6,620 single-copy orthologues. Amino acid and nucleotide sequences of the ortholog genes were aligned using MUSCLE (http://www.drive5.com/muscle/) [35]. All single copy ortholog alignments were concatenated into a super alignment matrix. A maximum likelihood-based phylogenetic tree was estimated based on the matrix of nucleotide sequences using RAxML (http://sco.h-its.org/exelixis/web/software/raxml/index.html.). Clade support was evaluated using the boot-strapping algorithm in the RAxML package with 100 alignment replicates. The constructed phylogenetic tree showed that hamster and other Cricetidae species were clustered closely first, and then clustered with Muridae, which is in agreement with their putative evolutionary relationships.

### The estimation of divergence time

The species divergence times were deduced with MCMCTree included in PAML (http://abacus.gene.ucl.ac.uk/software/paml.html) with the parameter set as “burn-in = 1,000,000, sample-number = 1,000,000sample-frequency = 10”.

### Gene family expansion and contraction analysis

To identify the expanded and contracted gene families, we performed analysis with CAFE (Computational Analysis of gene Family Evolution, https://sourceforge.net/projects/cafehahnlab/). We used Fisher’s exact test to detect pathway enrichment among the expanded and contracted genes (FDR < 0.05).

We obtained a new set of single-copy orthologues shared among four species *Mesocricetus auratus*, *Cricetulus griseus*, *Rattus norvegicus*, and *Mus musculus* to determine possible positively selected genes and genes under accelerated evolution. We implemented multiple alignment based on the protein sequences of single-copy orthologs with MUSCLE [35]. We estimated synonymous (Ks) and non-synonymous (Ka)s substitution rates by using the CODEML program from the PAML package [36]. We did a likelihood ratio test to identify genes under positive selection in the branch-site model. A total of 902 genes were identified as candidates for positively selected genes (*p*-value < 0.01, FDR < 0.05). Functional enrichment analysis of positively selected genes was performed based on Gene Ontology (GO, http://www.geneontology.org) and KEGG Pathway database (Kyoto Encyclopedia of Genes and Genomes, http://www.genome.jp/kegg).

### RNA sampling and sequencing

The 45 samples of the Syrian hamster were collected from 15 tissues and organs, including heart, liver, spleen, lung, kidney, pancreas, stomach, bowel, brain, muscle, testis, epididymis, lymph node, thymus and peripheral blood monocyte cells. Total RNA for transcriptome sequencing was extracted using NucleoSpin RNA kits (Macherey–Nagel). The quality including degradation and contamination was screened on 1% agarose gels. RNA integrity was evaluated by using the RNA Nano 6000 Assay Kit of the Agilent Bioanalyzer 2100 system (Agilent Technologies, CA, USA). 1.5 µg RNA per sample was obtained for library preparations. NEBNext® Ultra™ RNA Library Prep Kit for Illumina® (NEB, USA) was used for library preparation and library quality was assessed on the Agilent Bioanalyzer 2100 system. TruSeq PE Cluster Kit v3-cBot-HS (Illumina) was used for the clustering of the index-coded samples that was performed on a cBot Cluster Generation System. The libraries for each sample were sequenced on an Illumina NovaSeq 6000 platform and paired-end reads were generated. Raw data (raw reads) of fastq format were filtered by removing adapters, poly-N and the low-quality reads. As well as the Q20, Q30, GC-content and sequence duplication were calculated. Raw reads were submitted in Genbank (PRJNA662719, Table S2).

### Gene functional annotation

Gene function was annotated based on the public databases including Nr (NCBI non-redundant protein sequences), Nt (NCBI non-redundant nucleotide sequences), Pfam (Protein family), KOG/COG (Clusters of Orthologous Groups of proteins), Swiss-Prot (A manually annotated and reviewed protein sequence database), KO (KEGG Ortholog database). Gene Ontology (GO) enrichment analysis of the differentially expressed genes (DEGs) was implemented by the GOseq R package-based Wallenius non-central hyper-geometric distribution. After performing the differential expression analysis, we applied a p-value threshold of 0.05 to select genes that were statistically significantly differentially expressed between the drug-treated samples and the control samples. We used KOBAS software to test the statistical enrichment of differentially expressed genes in KEGG pathways [37]. The cluster of the annotation and KEGG enrichment analysis were conducted by DAVID bioinformatics Resources 6.8 and KOBAS-I [38–40].

### Transcriptome assembly

The transcriptomes of 15 tissues and organs were sorted and prepared for NCBI transcriptome shotgun assembly (TSA) submission, and the NCBI submission information is shown in Table S2. Reference genome and gene model annotation files were obtained from assembled genome and annotation in the present study. The index of the reference genome was built using HISAT2 v2.0.5 and paired-end clean reads were aligned to the reference genome using HISAT2 v2.0.5 [41]. The mapped reads of each sample were assembled by StringTie (v1.3.3b) in a reference-based approach [42]. StringTie uses a novel network flow algorithm as well as an optional de novo assembly step to assemble and quantitate full-length transcripts representing multiple splice variants for each gene locus.

featureCounts v1.5.0-p3 (http://bioinf.wehi.edu.au/featureCounts/) was used to count the read numbers mapped to each gene. FPKM of each gene was calculated based on the length of the gene and read counts mapped to this gene. FPKM, expected number of Fragments Per Kilobase of transcript sequence per Millions base pairs sequenced, considers the effect of sequencing depth and gene length for the reads count at the same time, and is currently the most commonly used method for estimating gene expression levels.

### Transcriptome quality and comparisons

Assembled transcriptome metrics showed an acceptable percentage (over 70%) of reads mapping back to each transcriptome indicating qualified assemblies. Bench-marking universal single-copy orthologs (BUSCO) v. 1.1.1 results using the nematode dataset (downloaded in March 2018) indicated that the transcriptomes have a moderate level of completeness (over 50%) [43, 44]. Cufflinks (V 2.2.1) analysis was run for the comparison of 15 different tissues and organs of hamster and different gene expression patterns were found Figure S2 [45].

### Distribution of alternative splicing modes

The annotation file of the hamster (gff3 file) was obtained from EVM. We performed an alternative splicing transcriptional landscape analysis on the Syrian hamster using the ASTALAVISTA web server (http://genome.crg.es/astalavista/).

### Western blotting

Tissues protein extracts were isolated from duodenum, small intestine, kidney, adrenal gland, colon, lung, esophagus, stomach, liver, heart, brain, spleen, testis and pancreas from wild-type Syrian hamsters and mice (n = 3) with RIPA lysis buffer (Beyotime Biotechnology, P0013C) containing 1% protease inhibitor cocktail solution (Roche, 04693132001). Bradford Protein Assay (CWBIO, Beijing, China) was used to estimate the protein concentration. 50 µg of protein prepared with loading buffer was separated in a 10% gel by SDS-PAGE and transferred to PVDF membranes (Millipore). After blocking for one hour at room temperature with a blocking solution of 5% milk, the membrane was incubated with primary antibody diluted in blocking solution at 4℃ overnight. For detection of the ACE2 protein, a 1:1,000 dilution of the ACE2 Rabbit monoclonal antibody (ET1611-58, HUABIO, China) was used as the primary antibody and a 1:10,000 dilution of the goat anti-rabbit antibody (ZB-5301, ZSBIO, China). GAPDH expression was demonstrated with a 1:10,000 dilution of GAPDH mouse monoclonal antibody (60,004–1-lg, Proteintech, USA) as the primary antibody and a 1:12,000 dilution of the goat anti-mouse antibody (ZB-5305, ZSBIO, China) as the secondary antibody.

### Gene enrichment analysis

In order to compare the genes enrichment of different tissues from human, Syrian hamster, mouse and rat, the main tissue differential expressing genes (top500, based on p value) of hamsters were identified first, then overlapped genes were screened out using Venny software for further gene clustering with R studio or GO (Gene Ontology) analysis with metascape software. To explore whether hamsters are superior in the field of cardiovascular research, 128 overlapped genes (in human, mouse and Syrian hamster) from 244 coronary artery disease (CAD) related orthologs were clustered in liver and heart tissues [22].

### SARS-CoV-2 pseudovirus assay

To generate a SARS-CoV2 pseudovirus, plasmids including lenti-Luciferase, psPAX and codon-optimized Spike with 18 amino acid deletion were co-transfected into 293 T cells following our previous study [46]. 1 × 10^4^ cells expressing ACE2 derived from human, mouse, rat and Syrian hamster were seeded into 96-well plates and infected with 50 μL SARS-CoV2 pseudovirus, and 72 h later, the relative luciferase activities were measured with the luciferase assay system (Promega) and GloMax Discover detector (Promega).

### SARS-CoV-2 infection assay

Syrian hamster kidney cancer cells HaK (Purchased from DSMZ) were seeded into 24-well plates, and infected with SARS-CoV-2 (isolated, BetaCoV/Wuhan/IVDC-HB-01/2020|EPI_ISL_402119) at MOI = 0.2. One hour later, medium was refreshed and virus genomes in cell supernatant at 24 or 72 h were detected using real-time RT-PCR (ORF1ab-foward primer CCCTGTGGGTTTTACACTTAA; ORF1ab-Forward reverse primer ACGATTGTGCATCAGCTGA; and the probe 5’-VIC-CCGTCTGCGGTATGTGGAAAGGTTATGG- BHQ1-3’). Total virus titres at 72 h in the virus-infected HaK cells were measured with Vero cell line as described previously [47]. For the immunofluorescence assay, HaK cells were seeded into Chambered Coverglass System (Thermo Scientific™ NuncTM Lab-Tek™) and infected with SARS-CoV2 at MOI = 10. 24 h later, cells were fixed, and virus detected with rabbit anti-SARS-CoV-2 polyclonal antibody as described previously [47]. All experiments were performed in biosafety level 3 laboratory.

### Statistical analysis

For the experimental data, data are presented as mean ± standard error of the mean (SEM). Unless otherwise stated, statistically significant differences between the two groups were analyzed by t-test when variances were equal, and 1-way analysis of variance followed by Dunnett test was used for multiple comparisons with Prism 7 (GraphPad Inc). P < 0.05 was statistically significant.

## Results

### Genome assembly of the Syrian hamster

The genome of the Syrian hamster is approximately 3.13Gbp, with a heterozygosity rate of ~ 0.22% (Table S4). 17-mer was predicted using k-mer distribution analysis and applied to genome assembly (Figure S1A). The assembled genome spanned 2.56 Gb with a scaffold N50 assembly size of 114.59 Mb and GC content of 41.79% based on 100.44 × Nanopore long reads and 110.91 × Hi-C linked reads (Table 1, Table S5). A total of 8,281 scaffolds covering 255.64 Mb (90.03%) of the genome were anchored and orientated into 22 pseudochromosomes ranging from 26.58 to 155.23 Mb (Figure S1B, Table S6). Around 95.80% completeness of the draft genome was evaluated by BUSCO (24) and CEGMA (25) (95.56% of the 248 core eukaryotic genes), which is the most complete assembled genome of the Syrian hamster (Table S7).

**Table 1:** Genome assembly and annotation of the Syrian hamster

Assembly		Annotation	
Assembly size (Gb)	2.31	Protein-coding Genes (Total predicted)	21,387
Genome size (Gb)	2.56	Protein-coding Genes (Total annotated)	21,193
Coverage	Illumina reads:98.25 × (307.53Gbp); Nanopore long reads: 100.44 × (314.37Gbp); Hi-C reads:110.91 × (336.06Gbp)	noncoding RNA	27,002
Contig N50 (bp)	35,650,180	microRNA	20,796
Number of contigs	8,600	tRNA	2,896
Scaffold N50 (bp)	114,594,012	rRNA	926
Number of scaffolds	8,303	snRNA	2,384
GC content	41.79%		

### Genomic annotation and architecture of the Syrian hamster

42.05% of the assembled genome from the Syrian hamster is composed of repetitive sequences (Table 2). The most frequent type of repeats are long terminal repeats (LTR), accounting for 32.45% of the genome sequence, while approximately 4.32% of the genome was identified as tandem repeats (Table 2). 21,387 protein-coding genes are predicted in the hamster genome, which is more similar to that of the Chinese hamster (21,298 genes) compared with other existing published rodent genomes (Table S1). In addition, the number of genes predicted in the present study shows almost 3,000 more than was annotated in 2013. Nearly 99.1% of genes (21,193) found in the Syrian hamster were functionally annotated and the lengths of the average gene and coding sequence (CDS) were predicted as 33,298.04 bp and 1,459.84 bp, respectively (Table 3). The results were consistent with the distribution of gene features in other rodents (Table S8). 20,796 miRNAs, 926 rRNAs, 2,384 snRNAs and 2,896 tRNAs were also found in the Syrian hamster to build a complete genome of this model (Table S9).

**Table 2:** Summary of statistics of annotated repeats in the genome of the Syrian hamster

A Sequence repeats in the genome of the Syrian hamster						
	Denovo + Repbase		TE proteins		Combined TEs	
	Length(bp)	Percent of sequence (%)	Length(bp)	Percent of sequence (%)	Length(bp)	Percent of sequence(%)
**DNA**	6,525,330	0.25	90,746	0	6,603,284	0.26
**LINE**	219,554,604	8.56	196,584,960	7.67	305,804,643	11.93
**SINE**	16,653,125	0.65	0	0	16,653,125	0.65
**LTR**	829,194,405	32.34	44,671,419	1.74	832,033,076	32.45
**Simple_repeat**	792	0	0	0	792	0
**Unknown**	25,350,712	0.99	0	0	25,350,712	0.99
**Total**	1,044,958,128	40.75	241,312,878	9.41	1,052,652,418	41.05
B Repeat sequences in the genome of the Syrian hamster						
**Type**	Repeat Size(bp)			% of genome		
**Trf**	110,690,172			4.32		
**Repeatmasker**	1,044,958,128			40.75		
**Proteinmask**	241,312,878			9.41		
**Total**	1,078,170,882			42.05

**Table 3:** Summary of statistics of predicted protein-coding genes

Gene set	Total number of gene	Average transcript length(bp)	Average CDS length(bp)	Average exons per gene	Average exon length(bp)	Average intron length(bp)
De novo						
Augustus	30,520	18,266.30	1,111.07	5.53	200.79	3,784.14
GlimmerHMM	383,542	5,555.94	427.93	2.73	156.64	2,960.91
SNAP	79,647	49,958.46	717.22	5.26	136.44	11,568.46
Geneid	40,070	29,193.85	991.51	5.27	188.1	6,603.06
Genscan	47,569	34,543.32	1,156.16	7	165.13	5,563.20
Homolog						
Cgr	37,537	13,070.07	1,142.64	5.17	220.99	2,859.99
Hsa	32,857	15,196.53	1,156.65	5.55	208.51	3,087.55
Mca	28,498	17,891.94	1,273.00	6.33	201.08	3,117.45
Mmu	28,128	18,200.02	1,298.26	6.43	201.95	3,113.36
Moc	28,394	18,066.77	1,282.80	6.39	200.62	3,111.46
Pma	27,570	18,297.88	1,361.89	6.58	206.83	3,032.59
Rno	36,489	14,692.09	1,238.03	5.42	228.22	3,040.73
RNAseq						
PASA	132,230	21,243.56	1,094.14	6.32	173.09	3,786.61
Cufflinks	106,114	31,794.03	3,604.75	7.44	484.53	4,377.43
EVM	37,268	17,477.57	1,023.02	5.46	187.24	3,686.28
Pasa-update	36,791	20,841.24	1,066.45	5.69	187.51	4,218.54
Final set	21,387	33,298.04	1,459.84	8.6	169.8	4,190.66

**Fig. 1:**
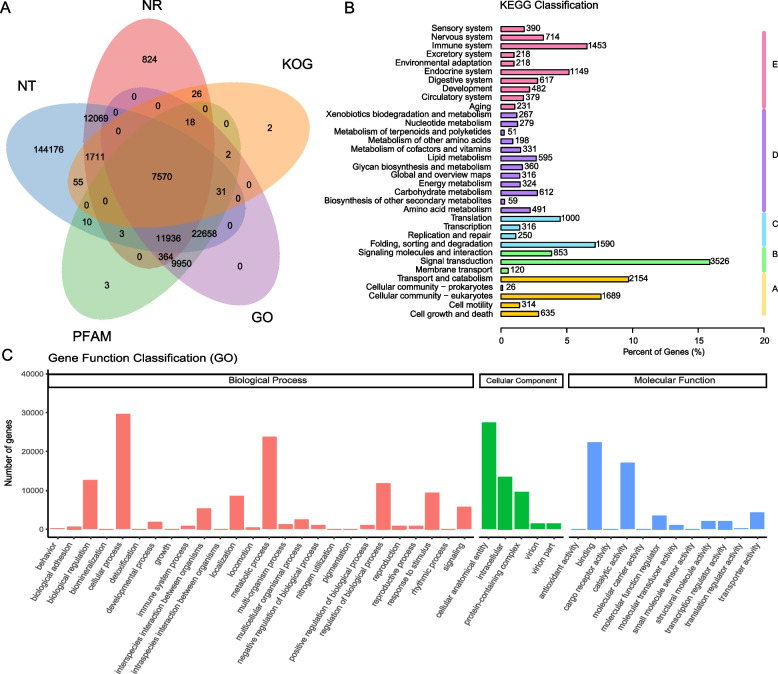
Gene annotation with transcriptomes of Syrian hamsters. **A** Gene function annotation in Protein databases (InterPro, Swiss-Prot, NR and KEGG) and Venn statistical results; **B** Gene function annotation predicted by KEGG pathway in the hamster. The vertical axis shows KEGG classification of metabolic pathways, the horizontal axis shows the number and percentage of genes annotated to the KEGG metabolic pathway. The genes are divided into five categories according to the KEGG metabolic pathway involved, A: Cellular Processes, B: Environmental Information Processing, C: Genetic Information Processing, D: Metabolism, E: Organismal Systems. **C** Gene function classification through gene orthology analysis. The horizontal axis shows GO Terms, and the vertical axis is the number of genes annotated to the GO Term

21,193 protein-coding genes in this study were screened in five functional protein databases (NR/NT, KOG, Pfam and GO) and we found that 99.10% of genes in the Syrian hamster could be predicted by searching with Augustus [29], PASA [48] and EVM [34] (Table S10). A total of 7570 genes were annotated from all these five databases (NR/NT, KOG, Pfam and GO) (Fig. 1a), and most of these genes were found to be involved in biological processes including metabolism and cellular processes (Fig. 1b and c).

### Identification of the phylogenetic position of Syrian hamster

**Fig. 2:**
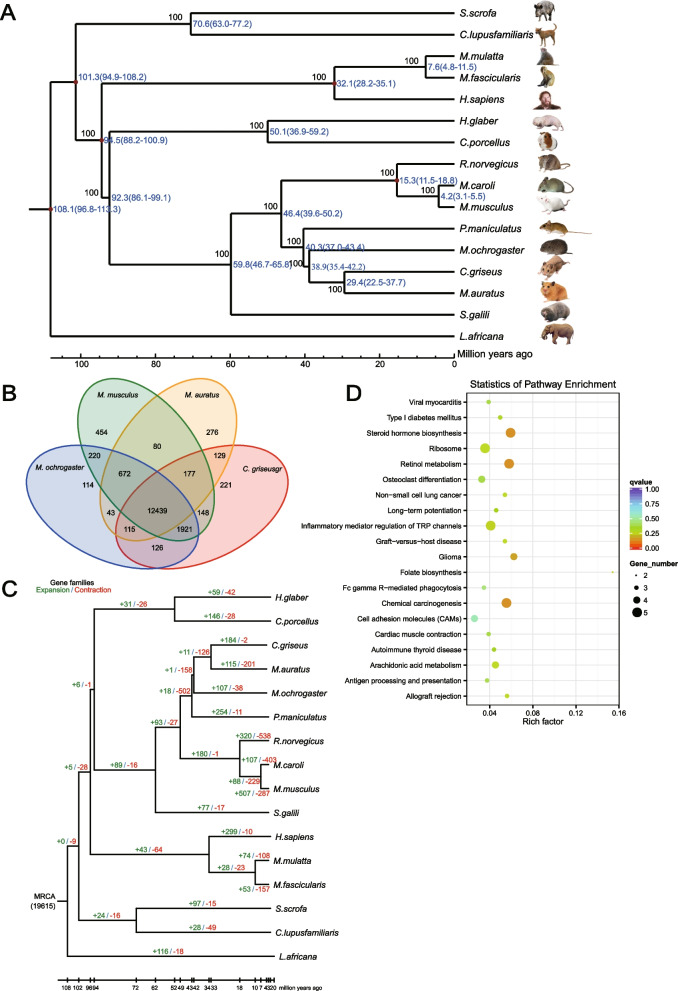
Comparative genomic analysis of *M.auratus*. **A** Consensus phylogenetic tree of *M.auratus* (Syrian hamster) and other mammalian species based on 6,620 single-copy genes. The divergence time are marked in each note with an error range. **B** Venn diagram of *M.auratus* gene families with *M. musculus*, *C. griseus* and *M. ochrogaster*. **C** Gene family expansions and contractions in the *M.auratus* genome. The numbers on each branch correspond to the numbers of gene families that have expanded (green) and contracted (red). MRCA: most recent common ancestor. **D** KEGG pathway enrichment of unique gene families in the *M.auratus* genome

To resolve the controversial issue regarding the origin of the Syrian hamster, 15 mammalian species closely related to the Syrian hamster that also have whole-genome sequences available in the public database (NCBI) were selected to reconstruct the phylogenetic tree (Fig. 2a). 19,615 gene families and 6,620 single-copy orthologues were identified in the Syrian hamster, which were also found in the other 15 mammalian species. Based on the single-copy orthologues, we reconstructed a phylogenetic tree with *Homo sapiens* as an exogenous species and *Loxodonta africana* as the outgroup. Chinese hamster (*Cricetulus griseus*) is most closely related to the Syrian hamster, diverging around 29.4 (22.5–37.7) million years ago. The speciation event of the ancestor of the Syrian hamster and *C. griseus* may have occurred 38.9 million years ago (Fig. 2a). We also compared the gene families of *C. griseus*, *Microtus ochrogaster*, *Mus musculus* and *Mesocricetus auratus*, and identified 276 gene families present only in the Syrian hamster. We identified 115 expanded gene families that refer to the cluster of genes duplicating during evolution and show 201 contracted gene families in the Syrian hamster, which might result from an accumulation of gene function loss with mutation (Figs. 2b and c). The genes associated with immune system pathways, metabolic pathways, carcinogenesis pathways and cardiac muscle pathways are over-represented in hamster-specific and expanded gene families compared with *C. griseus*, *M. ochrogaster*, *M. musculus*, each derived from the most recent common ancestor (Fig. 2d).

### Comparative transcriptomics of different tissues from the Syrian hamster

**Fig. 3:**
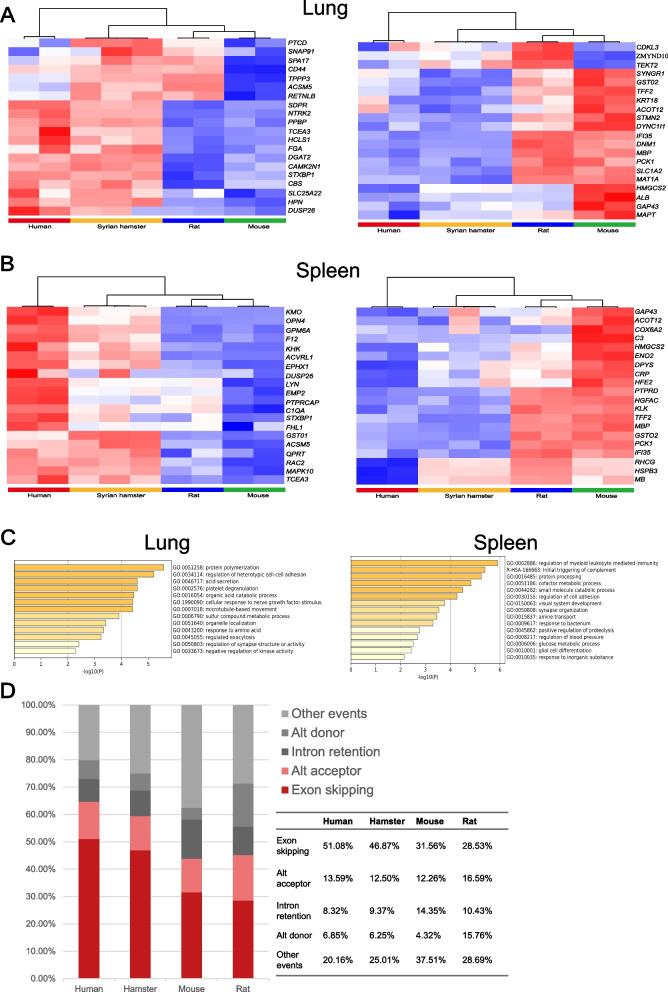
Transcriptome analysis of Syrian hamster. Heatmap of genes enriched for expression in lung (**A**) and spleen (**B**) of human, Syrian hamster, mouse and rat. The genes listed could be found in human, Syrian hamster, mouse and rat and are highly expressed. The top 500 differentially expressed genes in different hamster tissues were identified, then the 260 genes overlapping with GSE41464 (containing human, mouse and rat expression data) were used to generate heatmaps of lung and spleen tissues with the complete linkage cluster method. Left panel presents the genes upregulated in human lung or spleen and the corresponding genes expression in other species.Right panel presents the genes downregulated in human lung or spleen and the corresponding genes expression in other species. **C** GO (Gene Ontology) analysis of genes selected in lung and spleen using metascape software51. **D** An alternative splicing landscape analysis based on the transcriptomes of the human, hamster, mouse and rat

To dissect the landscape of gene expression profiles, a transcriptomic comparison from 15 tissues/organs of the Syrian hamster, including heart, liver, spleen, lung, kidney, pancreas, stomach, bowel, brain, muscle, testis, epididymis, lymph node, thymus and peripheral blood monocyte cells, was conducted (Figure S2). A total of 540,128 transcripts were assembled and 312,094 UniGenes were found in the Syrian hamster. Given that the lung is the primary infection target of SARS-COV-2 and recognizing the importance of the host immune system in the pathogenesis of SARS-COV-2 infection, a comparative transcriptomic analysis of lung and spleen tissues from human, mouse, rat and Syrian hamster was performed. The expression profiles of highly expressed genes in the Syrian hamster are more similar to human compared to mouse and rat, in particular, the genes associated with immune function and metabolism, including CRP (C-reactive protein), C3 (Complement C3), F12 (Coagulation factor XII), PCK1 (Phosphoenolpyruvate carboxykinase 1) and SLC1A2 (Solute carrier family 1 member 2) (Figs. 3a and b, Figure S2). Genes in the lung are enriched in several pathways including protein polymerization, acid secretion and platelet degranulation, while genes involved in myeloid leukocyte-mediated immunity and initial triggering of complement stand out in hamster spleen tissues (Fig. 3c).

Subsequently, an alternative splicing landscape analysis based on the transcriptomes of the human, hamster, mouse and rat was performed. The distribution of alternative splicing modes of the hamster shows that exon skipping in the hamster occurs at a rate of 48.87% and alternative exon usage is 12.50%, rates that are very similar to humans (47.69% exon skipping rate and 16.81% alternative exon usage), whereas in rat and mouse the exon skipping rates are 28.53% and 31.56% and alternative exon usage rates are 16.59% and 12.26% respectively (Fig. 3d).

### Characterization of the genes associated with human atherosclerosis in heart and liver of Syrian hamster

**Fig. 4:**
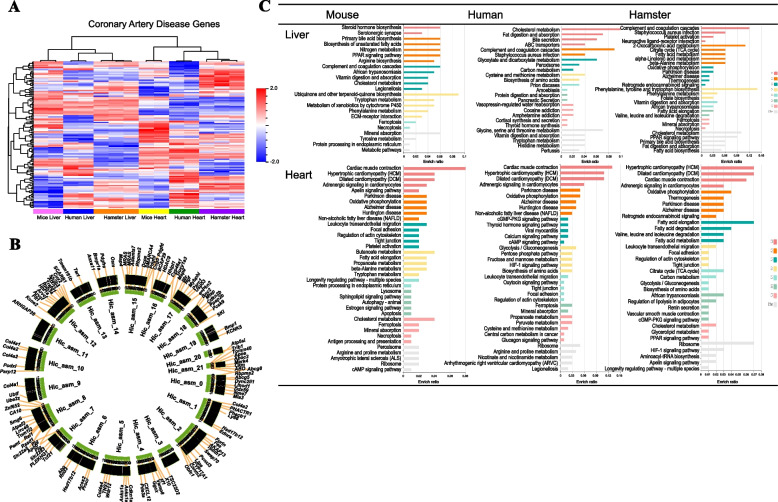
Transcriptomic analysis of the genes associated with CAD in Syrian hamster. **A** Heatmap of genes enriched for expression in liver and heart of human, Syrian hamster and mouse. The genes listed could be found in human, Syrian hamster, mouse are highly expressed. The top 500 differentially expressed genes in different hamster tissues were identified based on p value and the 126 genes overlapping with GSE41464 (containing human and mouse expression data) were used to generate the heatmaps of heart with the complete linkage cluster method. **B** Schematic representation of the Syrian hamster chromosomes together with the positions of 126 genes of Syrian hamster found associated with human CAD based on human CAD GWAS candidate genes. **C** The KEGG enrichment ratio of the top 50 highly expressed genes from the liver and heart of Syrian hamster, human and mouse

The gene expression pattern of human liver and heart is more similar to the Syrian hamster compared to mouse, which shows down-regulation or up-regulation in the same gene clusters (Fig. 4a). Based on the transcriptomic data of the liver of the Syrian hamster, we employed DAVID [49] to detect the pathways associated with the most highly expressed genes and found multiple pathways involved in human coronary artery disease (CAD) including the PI3K-Akt signalling, insulin resistance, chemokine signalling, fat digestion and the adipocytokine pathways. By comparing with human CAD GWAS (genome wide association studies) candidate genes, we found 126 genes matched the Syrian hamster and mapped them to its chromosomal structure (Fig. 4b). Focusing on key genes associated with human CAD, we found that humans and the Syrian hamster have one isoform of the brain natriuretic peptide (NPPB) gene while mice have three and rats have two. According to AGTR1 (angiotensin II receptor type I) conservation and divergence data, hamster isoform-3.625 is more similar to human AGTR1, while hamster isoform-1.639 is more similar to mouse/rat Agtr1a. We also identified CETP (cholesterol ester transfer protein) in the Syrian hamster with a similar function to humans while this gene is absent from both mouse and rat. KEGG functional enrichment analysis of the top 50 genes expressed in liver and heart of hamster, mouse and human demonstrates that they share similar functional pathways, while the HIF-1 (hypoxia inducible factor) signalling pathway was significantly enriched both in human and hamster (Fig. 4c).

### Characterization of Syrian hamster genes that are involved in SARS-COV-2 infection

**Fig. 5:**
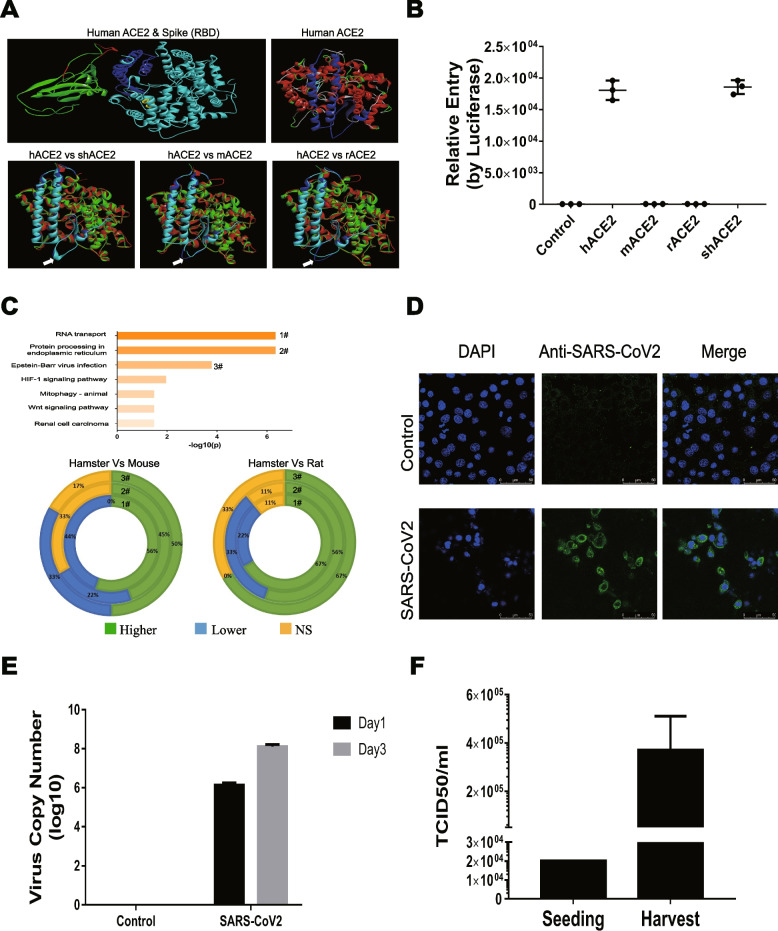
Characterization of Syrian hamster ACE2 receptor and other genes involved in SARS-COV-2 infection. **A** Interaction model between human ACE2 (cyan) and SARS-CoV spike RBD (green) (upper left panel, Protein Data Bank (PDB) accession number: 6m0j). The key binding site on ACE2 is marked in blue (upper right panel). The 3D structures of ACE2 from hamster, mouse and rat were predicted using GalaxyTBM and compared with human ACE2 by 3D-Match, and key binding site on ACE2 is marked in blue (human) or cyan (lower panels). The white arrows show potential key loop sites with different structures. **B** HEK-293 T cells stably expressing ACE2 from human, Syrian hamster, rat and mouse were infected with SARS-CoV-2 pseudovirus and luciferase activities were measured using the Luciferase Assay System. **C** The interaction of 65 reported drugable genes were analyzed with String Software and KEGG pathway enrichment is presented (upper panel). The top three KEGG pathway-related genes in human, mice or hamster were aligned with MegAlign. The percentage of hamster-derived genes with higher, lower or no significant difference (Ns) homology to human are marked green, blue or yellow respectively. **D** Syrian hamster transformed kidney epithelial cells (HaK) were infected with SARS-CoV2, then viral protein was detected using SARS-CoV2 polyclonal antibody by immunofluorescence assay. **E** Syrian hamster cells (HaK) were infected with SARS-CoV2 at a multiplicity of infection (MOI) of 0.2. One hour later, medium was refreshed and virus genomes in cell supernatant at 24 or 72 h were detected by quantitative real-time RT-PCR. **F** HaK cells were infected with SARS-CoV2 (as forE) and cell supernatant was harvested at 72 h post-infection, virus titre was determined using the Vero cell line

To reveal whether there are genetic advantages of the Syrian hamster as an animal model for COVID-19, several genes involved in SARS-COV-2 infection were further characterized. Studies have proven that Spike (S) protein in both SARS-CoV and SARS-CoV-2 engages the human angiotensin-converting enzyme 2 (hACE2) as a cellular receptor for entry and infection [50]. We compared the convergence and divergence of ACE2 among human, Syrian hamster, mouse and rat (Figure S3a) and found that Syrian hamster ACE2 has a higher homology with human (84.5%) compared with rat (82.5%) and mouse (82.1%). ACE2 expression in different organs and tissues exhibits a similar pattern among humans and the three rodents, enriched in the gastrointestinal tract, kidneys and adrenal glands (Figures S3b, d, e and f). The potential binding affinity of the variants of the key amino acids of ACE2 variations from different species and the spike receptor-binding domain (RBD) of SARS-CoV-2 was evaluated using public deep mutagenesis data [51]. As shown in Figures S3c and S4a, both mouse and rat showed several variations, especially at the K353 site, which could weaken RBD-binding capacity, while the binding site of hamster ACE2 for SARS-COV-2 RBD is almost the same as human ACE2. The structure of hamster ACE2 also indicated that the key region that interacts with RBD is more similar to human compared to the other rodents (Fig. 5a). SARS-CoV-2 pseudo-virus assays further proved that the ACE2 receptor derived from humans and hamsters could efficiently mediate SARS-CoV-2 entry, whereas the cells expressing ACE2 from rat or mouse (Figure S4b) were not infected by the pseudo-virus (Fig. 5b).

The S protein of SARS-CoV-2 is enzymatically processed by host serine proteases such as Furin and TMPRSS2, an essential process for efficient fusion and release of the virus contents into the host cell cytosol [50, 52]. Homology analysis demonstrated that Furin is more highly conserved (> 90%) than TMPRSS2 (< 80%), but TMPRSS2 proteins derived from humans, mouse and hamster are all able to effectively cleave S (Figure S5), indicating that the S protein cleavage process may not be the main factor restricting SARS-CoV-2 infection in different animal models. After internalization, virus replication and progeny release are predominately dependent on host cell factors. The interaction spectrum between SARS-CoV-2 proteins and host factors has been reported, showing 332 high-confidence protein–protein interactions between SARS-CoV-2 and human proteins and revealing 65 druggable genes [53]. Protein interaction analysis using STRING software [54] demonstrated that these 65 druggable genes are enriched in several pathways including RNA transport, protein processing in endoplasmic reticulum and EBV (Epstein-Barr virus) infection. Interestingly, further homologous alignments show that in the top three key signalling pathways, more hamster-derived genes than rat or mouse-derived genes have higher homology to human genes (Fig. 5c). Importantly, after SARS-COV-2 virus infection of Syrian hamster kidney transformed epithelial cells (HaK), which express high levels of ACE2 (Figure S4b), viral protein was highly expressed (Fig. 5d), virus genome number was increased over a time course (Fig. 5e) and more progeny virus were produced (Fig. 5f), suggesting that Syrian hamster cells fully support SARS-COV-2 infection and replication.

## Discussion

The genome assembly of the Syrian hamster presented in this study is more complete and provides more information than the previous version (Table S1) [55–57]. Additionally, transcriptomes derived from 15 organs and tissues of the hamster were analyzed, which improves our functional understanding of the model and permits more comprehensive and robust phylogenetic comparisons with other species. The power of the current study is exemplified by the elucidation of 21,193 protein-coding genes, whereas previous studies have annotated only 18,257 genes (Fig. 1, Table 1, Table S1), although we appreciate that further research efforts are needed to achieve a fully integrated functional annotation of the complex regions of the hamster genome in combination with transcriptomic profiles and proteomics in all organs.

Our transcriptomic studies show that the number of genes expressed in the Syrian hamster is higher than those in mouse and rat, despite the similarity of genome sizes between the rodents, suggesting that the Syrian hamster may adopt a more complex expression strategy in coding genes. We also revealed that Syrian hamsters share a highly similar gene expression pattern with humans and, interestingly, the expression profiles of highly expressed genes in the hamster’s heart, lung and spleen are aligned more closely with human expression patterns compared to the mouse/rat-human expression profiles. Moreover, the splicing modes of the transcriptomic landscape in the Syrian hamster show similarities with humans, allowing for wider gene expression profiles than noted for mice and rats [58]. It has been reported that splicing, one of the central cellular pathways, is essential for SARS-CoV-2 replication [59]. Alternative splicing (AS) provides a resource for modulation of gene coding in eukaryotic cells and regulates the expression of functional genes in reaction to environmental changes [60]. Our analysis of AS across the Syrian hamster, human, rat and mouse suggests the Syrian hamster is a more suitable small animal model for accurately assessing the interaction between SARS-CoV-2 and host cells, as the AS patterns are more closely conserved between humans and Syrian hamsters.

Recently, the proteomics of SARS-CoV-2-infected host cells demonstrated that viral infection could reshape host cell gene splicing and protein homeostasis [59]. To further demonstrate the suitability of the Syrian hamster as an animal model for COVID-19, we characterized the genes that are involved in SARS-COV-2 infection. We extracted the amino acid sequence and protein structure of Syrian hamster ACE2 (shACE2) and found that shACE2 shows higher homology to hACE2 compared to 14 other species including mouse and rat. Most importantly, the results demonstrated that shACE2 RBD amino acid sequence is highly related to the human sequence and functionally results in SARS-COV-2 infection, (Fig. 5b), while mouse ACE2 and rat ACE2 did not support SARS-COV-2 infection [53]. Further genetic analysis demonstrated that in the top three key signalling pathways involved in SARS-COV-2 infection, more hamster-derived genes presented higher homology with human genes than those found in mouse or rat (Fig. 5 c). Collectively, this study, along with others, demonstrates that the Syrian hamster will be an appropriate animal model for understanding SARS-CoV-2 pathogenesis, testing vaccines and developing antiviral drugs. Of note, the vast majority of wild-type hamsters did not die upon SARS-CoV-2 infection, which is consistent with human infections. It would be conceivable and important to develop COVID-19 hamster models with comorbidities such as immune-deficiency, diabetes mellitus, hypertension, or obesity [7]. To this end, the recent well-developed transgenic Syrian hamster platform and disease models [61, 62] may provide powerful research tools. Indeed, a study by some of us in using *STAT2* knockout Syrian hamsters (*STAT2*^−/−^) has demonstrated that STAT2 signalling plays a dual role in viral infections, both aggravating lung injury and also limiting the systemic spread of SARS-CoV-2 [63]. Similarly, the use of *RAG2* knockout (*RAG2*^−/−^) and *IL2RG* knockout (*IL2RG*^−/−^) Syrian hamsters developed by us provided great insights into the role adaptive immunity and natural killer cells in host defence against SARS-CoV-2 infection [64].

The majority of CVD is attributed to atherosclerosis, characterized by endothelial dysfunction, chronic inflammation, dyslipidemia, and accumulation of lipid in arterial walls. Generally, non-genetically modified rats and mice are not suitable for studying diet-induced changes in plasma lipid and lipoprotein concentrations and the development of atherosclerotic lesions, because they do not form the aortic lesions or changes seen in humans [16]. Since the 1980s hamsters have been used as an animal model to assess diet-induced atherosclerosis [65]. The Syrian hamster is considered more desirable because of its low endogenous cholesterol synthesis rate, receptor-mediated uptake of low-density lipoprotein cholesterol, the presence of cholesterol ester transfer protein (CETP) activity [66] and the uptake of most LDL-C through the LDL receptor pathway [65]. Increasing number of studies show that the physiological and metabolic characteristics and genetic background of the Syrian hamster are similar to humans, which suggests it as an advantageous model for revealing the mechanisms of cardiovascular disease at multiple levels [67, 68]. Unlike mouse, cholesterol ester transfer protein (CETP), which plays an important role in regulating lipid metabolism in humans [56], has been found to be highly expressed in the Syrian hamster. The apolipoprotein B (apoB) mRNA editing activity is found more abundantly in the small intestine rather than the liver in both humans and hamsters, and apoB-48 is only expressed in small intestine of the Syrian hamsters [69]. Additionally, the Syrian hamster is prone to coronary artery stenosis, occlusion, premature death and other cardiovascular diseases. Based on CRISPR/cas9 gene editing technology, the LDLR gene knockout golden hamster showed a similar disease phenotype to familial hypercholesterolemia (FH) patients. It has been found that the blood lipid level of the hybrid golden hamster was significantly increased in normal dietary conditions and the hypercholesterolemia mainly increased LDLC in a short-term hypercholesterolemia/high-fat diet. Typical lesions appeared in both aorta and coronary arteries, resulting in pathology such as myocardial infarction, vascular stenosis and occlusion. The homozygous LDLC KO golden hamster also showed similar but more severe phenotypic characteristics, as well as premature death. These results reflect that the model can effectively simulate the characteristics of human FH disease [17]. Together these factors demonstrate that the Syrian hamster may be the most suitable and effective animal model to simulate the characteristics of human lipid metabolism and related cardiovascular diseases. Moreover, its larger litter and body sizes in comparison to mouse and rat (litter size) models renders it more convenient for experimental operation and sampling.

## Conclusions

This study provides a comprehensive analysis of the genome and transcriptome of the Syrian hamster, demonstrating that Syrian hamsters more closely resemble humans in terms of gene expression patterns and alternative splicing when compared to rats and mice. We have previously demonstrated that certain key human cytokines, but not murine cytokines, are functional in Syrian hamster tumor models, demonstrating the breadth of application of this model [70, 71]. The data presented here also provides new biological insights and knowledge about the *M. auratus* species that will improve its application in medical research. Future studies should consider expanding this study to get additional essential data of other important tissues, determining functional similarities between the Syrian hamster and human. Of note, the lack of research tools represents a major barrier to effective use of Syrian hamster models. Immunologic reagents for examining host immune response and particular gene expression, and transgenic disease models will all be required for a more complete evaluation of the value of this model.

## Supplementary Information

Supplementary Material 1.

Supplementary Material 2.

## Data Availability

The genome assembly data were deposited in NCBI (PRJNA666008). A total of 540,128 transcripts were assembled by combining all data and 312,094 unigenes were found in the Syrian hamster and deposited in NCBI BioProject PRJNA662719. Transcription profiling of different tissues from human, mouse, and rat were downloaded from GEO database (GSE41464). Metadata of mouse and human were obtained from the Mouse Genome Informatics and GTEX. All the materials developed in this study are available.
