# Supplementary material for: Genomic-transcriptomic analysis identifies the Syrian hamster as a superior animal model for human diseases
Source: BMC Genomics. 2025 Mar 24;26:286. doi: 10.1186/s12864-025-11393-4 (PMC11931762; doi:10.1186/s12864-025-11393-4)
Supplement: Supplementary file 1 — Supplementary Material 1. [file 12864_2025_11393_MOESM1_ESM.docx]

Table S 1 Genome assembly statistics of Syrian hamster

|  | Syrian hamster  MesAur2.0 -2020 | Syrian hamster  MesAur1.0 | Chinese hamster  CriGri_1.0 |
| --- | --- | --- | --- |
| Assembly size (Gb) | 2.31 | 2.07 | 2.32 |
| Genome size (Gb) | 2.57 | 2.50 | 2.40 |
| Chromosomes | 44 (male) | 44(female) |  |
| Sequencing technology | Illumina;Nanopore;Hi-C | Illumina HiSeq | Illumina GA IIx |
| Coverage | 100×Illumina;  100×Nanopore;  100×Hi-C | 115×Illumina | 130×Illumina |
| Assembler | wtdbg2 | allpaths v. R44683 | SOAPdenovo v. 1.05 |
| Contig N50 (bp) | 35,650,180 | 22,511 | 39,361 |
| Number of contigs | 8,600 | 237,700 | 265,787 |
| Scaffold N50 (bp) | 114,594,012 | 12,753,307 | 1,147,233 |
| Number of scaffolds | 8,303 | 21,484 | 109,152 |
| Total annotated gene | 21,193 | 18,257 | 21,298 |

Table S 2 BioSample information of BioProject PRJNA662719 a deep transcriptome sequencing of the Syrian hamster

| Accession | Sample Name | SPUID | Tax ID | Strain | library_ID | title |
| --- | --- | --- | --- | --- | --- | --- |
| SAMN16093387 | heart1 | heart1 | 10036 | LVG(syr) | FRAS202085839-1a | RNA-Seq of Syrian hamster: adult male heart |
| SAMN16093388 | heart2 | heart2 | 10036 | LVG(syr) | FRAS202085840-1a | RNA-Seq of Syrian hamster: adult male heart |
| SAMN16093389 | heart3 | heart3 | 10036 | LVG(syr) | FRAS202085841-1a | RNA-Seq of Syrian hamster: adult male heart |
| SAMN16093390 | liver1 | liver1 | 10036 | LVG(syr) | FRAS202085842-1a | RNA-Seq of Syrian hamster: adult male liver |
| SAMN16093391 | liver2 | liver2 | 10036 | LVG(syr) | FRAS202085843-1a | RNA-Seq of Syrian hamster: adult male liver |
| SAMN16093392 | liver3 | liver3 | 10036 | LVG(syr) | FRAS202085844-1a | RNA-Seq of Syrian hamster: adult male liver |
| SAMN16093393 | spleen1 | spleen1 | 10036 | LVG(syr) | FRAS202085845-1a | RNA-Seq of Syrian hamster: adult male spleen |
| SAMN16093394 | spleen2 | spleen2 | 10036 | LVG(syr) | FRAS202085846-1a | RNA-Seq of Syrian hamster: adult male spleen |
| SAMN16093395 | spleen3 | spleen3 | 10036 | LVG(syr) | FRAS202085847-1a | RNA-Seq of Syrian hamster: adult male spleen |
| SAMN16093396 | lung1 | lung1 | 10036 | LVG(syr) | FRAS202085848-1a | RNA-Seq of Syrian hamster: adult male lung |
| SAMN16093397 | lung2 | lung2 | 10036 | LVG(syr) | FRAS202085849-1a | RNA-Seq of Syrian hamster: adult male lung |
| SAMN16093398 | lung3 | lung3 | 10036 | LVG(syr) | FRAS202085850-1a | RNA-Seq of Syrian hamster: adult male lung |
| SAMN16093399 | kidney1 | kidney1 | 10036 | LVG(syr) | FRAS202085851-1a | RNA-Seq of Syrian hamster: adult male kidney |
| SAMN16093400 | kidney2 | kidney2 | 10036 | LVG(syr) | FRAS202085852-1a | RNA-Seq of Syrian hamster: adult male kidney |
| SAMN16093401 | kidney3 | kidney3 | 10036 | LVG(syr) | FRAS202085853-1a | RNA-Seq of Syrian hamster: adult male kidney |
| SAMN16093402 | pancre1 | pancre1 | 10036 | LVG(syr) | FRAS202096902-1r | RNA-Seq of Syrian hamster: adult male pancreas |
| SAMN16093403 | pancre2 | pancre2 | 10036 | LVG(syr) | FRAS202096903-1r | RNA-Seq of Syrian hamster: adult male pancreas |
| SAMN16093404 | pancre3 | pancre3 | 10036 | LVG(syr) | FRAS202096904-1r | RNA-Seq of Syrian hamster: adult male pancreas |
| SAMN16093405 | stomac1 | stomac1 | 10036 | LVG(syr) | FRAS202085857-1a | RNA-Seq of Syrian hamster: adult male stomach |
| SAMN16093406 | stomac2 | stomac2 | 10036 | LVG(syr) | FRAS202085858-1a | RNA-Seq of Syrian hamster: adult male stomach |
| SAMN16093407 | stomac3 | stomac3 | 10036 | LVG(syr) | FRAS202085859-1a | RNA-Seq of Syrian hamster: adult male stomach |
| SAMN16093408 | bowel1 | bowel1 | 10036 | LVG(syr) | FRAS202085860-1a | RNA-Seq of Syrian hamster: adult male bowel |
| SAMN16093409 | bowel2 | bowel2 | 10036 | LVG(syr) | FRAS202085861-1a | RNA-Seq of Syrian hamster: adult male bowel |
| SAMN16093410 | bowel3 | bowel3 | 10036 | LVG(syr) | FRAS202085862-1a | RNA-Seq of Syrian hamster: adult male bowel |
| SAMN16093411 | brain1 | brain1 | 10036 | LVG(syr) | FRAS202085863-1a | RNA-Seq of Syrian hamster: adult male brain |
| SAMN16093412 | brain2 | brain2 | 10036 | LVG(syr) | FRAS202085864-1a | RNA-Seq of Syrian hamster: adult male brain |
| SAMN16093413 | brain3 | brain3 | 10036 | LVG(syr) | FRAS202085865-1a | RNA-Seq of Syrian hamster: adult male brain |
| SAMN16093414 | muscle1 | muscle1 | 10036 | LVG(syr) | FRAS202085866-1a | RNA-Seq of Syrian hamster: adult male muscle |
| SAMN16093415 | muscle2 | muscle2 | 10036 | LVG(syr) | FRAS202085867-1a | RNA-Seq of Syrian hamster: adult male muscle |
| SAMN16093416 | muscle3 | muscle3 | 10036 | LVG(syr) | FRAS202085868-1a | RNA-Seq of Syrian hamster: adult male muscle |
| SAMN16093417 | testis1 | testis1 | 10036 | LVG(syr) | FRAS202085869-1a | RNA-Seq of Syrian hamster: adult male testis |
| SAMN16093418 | testis2 | testis2 | 10036 | LVG(syr) | FRAS202085870-1a | RNA-Seq of Syrian hamster: adult male testis |
| SAMN16093419 | testis3 | testis3 | 10036 | LVG(syr) | FRAS202085871-1a | RNA-Seq of Syrian hamster: adult male testis |
| SAMN16093420 | epidid1 | epidid1 | 10036 | LVG(syr) | FRAS202085872-1a | RNA-Seq of Syrian hamster: adult male epididymis |
| SAMN16093421 | epidid2 | epidid2 | 10036 | LVG(syr) | FRAS202085873-1a | RNA-Seq of Syrian hamster: adult male epididymis |
| SAMN16093422 | epidid3 | epidid3 | 10036 | LVG(syr) | FRAS202085874-1a | RNA-Seq of Syrian hamster: adult male epididymis |
| SAMN16093423 | lymphN1 | lymphN1 | 10036 | LVG(syr) | FRAS202085875-1a | RNA-Seq of Syrian hamster: adult male lymph node |
| SAMN16093424 | lymphN2 | lymphN2 | 10036 | LVG(syr) | FRAS202085876-1a | RNA-Seq of Syrian hamster: adult male lymph node |
| SAMN16093425 | lymphN3 | lymphN3 | 10036 | LVG(syr) | FRAS202096901-1r | RNA-Seq of Syrian hamster: adult male lymph node |
| SAMN16093426 | thymus1 | thymus1 | 10036 | LVG(syr) | FRAS202085878-1a | RNA-Seq of Syrian hamster: adult male thymus |
| SAMN16093427 | thymus2 | thymus2 | 10036 | LVG(syr) | FRAS202085879-1a | RNA-Seq of Syrian hamster: adult male thymus |
| SAMN16093428 | thymus3 | thymus3 | 10036 | LVG(syr) | FRAS202085880-1a | RNA-Seq of Syrian hamster: adult male thymus |
| SAMN16093429 | blood1 | blood1 | 10036 | LVG(syr) | FRAS20H100696-1b | RNA-Seq of Syrian hamster: adult male blood |
| SAMN16093430 | blood2 | blood2 | 10036 | LVG(syr) | FRAS20H100697-1r | RNA-Seq of Syrian hamster: adult male blood |
| SAMN16093431 | blood3 | blood3 | 10036 | LVG(syr) | FRAS20H100698-1r | RNA-Seq of Syrian hamster: adult male blood |

Table S 3 15 mammalian species that are phylogenetic closely related to Syrian hamsters

| Scientific name | Asembly Version | Lineage( abbreviated ) |
| --- | --- | --- |
| *Mesocricetus auratus* | MesAur2.0 -2020 | Euarchontoglires; Glires; Rodentia; Myomorpha; Muroidea; Cricetidae; Cricetinae; Mesocricetus |
| *Cricetulus griseus* | ncbi.all.GCF_000223135.1 | Euarchontoglires; Glires; Rodentia; Myomorpha; Muroidea; Cricetidae; Cricetinae; Cricetulus |
| *Microtus ochrogaster* | ncbi.all.GCF_000317375.1 | Euarchontoglires; Glires; Rodentia; Myomorpha; Muroidea; Cricetidae; Arvicolinae; Microtus |
| *Heterocephalus glaber* | GCA_000247695.1 | Euarchontoglires; Glires; Rodentia; Hystricomorpha; Bathyergidae; Heterocephalus |
| *Peromyscus maniculatus* | ncbi.all.GCF_000500345.1 | Euarchontoglires; Glires; Rodentia; Myomorpha; Muroidea; Cricetidae; Neotominae; Peromyscus |
| *Rattus norvegicus* | ncbi.all.GCF_000001895.5 | Euarchontoglires; Glires; Rodentia; Myomorpha; Muroidea; Muridae; Murinae; Rattus |
| *Mus musculus* | ncbi.all.GCF_000001635.26 | Euarchontoglires; Glires; Rodentia; Myomorpha; Muroidea; Muridae; Murinae; Mus; Mus |
| *Mus caroli* | ncbi.all.GCF_900094665.1 | Euarchontoglires; Glires; Rodentia; Myomorpha; Muroidea; Muridae; Murinae; Mus; Mus |
| *Cavia porcellus* | ncbi.all.GCF_000151735.1 | Euarchontoglires; Glires; Rodentia; Hystricomorpha; Caviidae; Cavia |
| *Spalax galili* | ncbi.all.GCF_000622305.1 | Euarchontoglires; Glires; Rodentia; Myomorpha; Muroidea; Spalacidae; Spalacinae; Nannospalax |
| *Sus scrofa* | GCA_000003025.6 | Laurasiatheria; Artiodactyla; Suina; Suidae; Sus |
| *Canis lupus familiaris* | GCA_000002285.2 | Laurasiatheria; Carnivora; Caniformia; Canidae; Canis |
| *Loxodonta africana* | GCA_000001905.1 | Afrotheria; Proboscidea; Elephantidae; Loxodonta |
| *Macaca mulatta* | GCA_003339765.3 | Euarchontoglires; Primates; Haplorrhini; Catarrhini; Cercopithecidae; Cercopithecinae; Macaca |
| *Macaca fascicularis* | GCA_000364345.1 | Euarchontoglires; Primates; Haplorrhini; Catarrhini; Cercopithecidae; Cercopithecinae; Macaca |
| *Homo sapiens* | ncbi.all.GCF_000001405.38 | Euarchontoglires; Primates; Haplorrhini; Catarrhini; Hominidae; Homo |

Table S 4 Estimation of genome size based on 17-mer statistics

| **Kmer** | **Depth** | **n_kmer** | **Genome_size(M)** | **Revised Genome_size(M)** | **Heterozygous_rate(%)** | **Repeat_rate(%)** |
| --- | --- | --- | --- | --- | --- | --- |
| 17 | 50 | 157,769,234,231 | 3155.38 | 3130.53 | 0.22 | 64.65 |

Table S 5 Sequencing statistics from the Oxford Nanopore PromethION platform

| Active channels | Mean read length (kb) | Mean read quality | Median read length (kb) | Median read quality | Number of reads | Read length N50 (kb) | Total bases (Gb) |
| --- | --- | --- | --- | --- | --- | --- | --- |
| 2892 | 21.7 | 7.2 | 20.3 | 8.6 | 14486888 | 36.9 | 314.37 |

Table S 6 Statistics of chromosome length disctribution based on clustered scaffolds

| **Chromosome-ID** | **Cluster_Number** | **3d_dna_Length (bp)** |
| --- | --- | --- |
| Hic_asm_1 | 4 | 104,684,488 |
| Hic_asm_2 | 19 | 134,003,719 |
| Hic_asm_3 | 28 | 153,847,731 |
| Hic_asm_4 | 5 | 85,223,946 |
| Hic_asm_5 | 16 | 96,694,069 |
| Hic_asm_6 | 16 | 155,226,236 |
| Hic_asm_7 | 10 | 151,752,808 |
| Hic_asm_8 | 71 | 92,255,657 |
| Hic_asm_9 | 6 | 130,305,181 |
| Hic_asm_10 | 43 | 125,440,525 |
| Hic_asm_11 | 27 | 122,959,895 |
| Hic_asm_12 | 3 | 116,903,082 |
| Hic_asm_13 | 4 | 116,852,966 |
| Hic_asm_14 | 5 | 108,597,311 |
| Hic_asm_15 | 5 | 107,512,938 |
| Hic_asm_16 | 8 | 114,594,012 |
| Hic_asm_17 | 6 | 84,204,844 |
| Hic_asm_18 | 4 | 83,864,794 |
| Hic_asm_19 | 3 | 83,671,825 |
| Hic_asm_20 | 17 | 80,772,012 |
| Hic_asm_21 | 2 | 32,448,689 |
| Hic_asm_22 | 17 | 26,579,874 |
| Scaffold | 8281 | 255,639,780 |

Table S 7 Assessment of CEGMA and BUSCOs in the mau genome.

| CEGMA |  |  | BUSCOS |  |
| --- | --- | --- | --- | --- |
| complete |  |  |  | Percentage (%) |
| # Prots | 231 |  | Complete BUSCOs | 95.8 |
| %completeness | 93.15 |  | Complete and single-copy BUSCOs | 94.7 |
| complete + partial |  |  | Complete and duplicated BUSCOs | 1.1 |
| # Prots | 237 |  | Fragmented BUSCOs | 2.1 |
| %completeness | 95.56 |  | Missing BUSCOs | 2.1 |
| core genes dataset | 248 most highly-conserved CEGs |  | Total BUSCO groups searched | 4104 |

Table S 8 Summary statistics of gene sets compared with other species

| Species | Number | Average transcript length(bp) | Average CDS length(bp) | Average exons per gene | Average exon length(bp) | Average intron length(bp) |
| --- | --- | --- | --- | --- | --- | --- |
| Cgr | 21,298 | 29,969.43 | 1,560.21 | 8.93 | 174.71 | 3,582.37 |
| Hsa | 22,693 | 46,731.20 | 1,721.97 | 9.75 | 176.66 | 5,145.56 |
| Mca | 21,050 | 38,567.02 | 1,655.17 | 9.26 | 178.81 | 4,470.57 |
| Mmu | 23,565 | 37,082.87 | 1,642.15 | 9.06 | 181.3 | 4,398.29 |
| Moc | 20,077 | 39,423.96 | 1,675.95 | 9.55 | 175.58 | 4,417.50 |
| Pma | 21,608 | 38,366.74 | 1,637.24 | 9.21 | 177.86 | 4,476.42 |
| Rno | 22,926 | 38,372.65 | 1,611.87 | 9.04 | 178.35 | 4,573.48 |
| Mau* | 21,387 | 33,298.04 | 1,459.84 | 8.6 | 169.8 | 4,190.66 |

Table S 9 summary statistic of noncoding RNA

| Type | Copy number (w*) | Average length(bp) | Total length(bp) | Percentage (%) of genome |
| --- | --- | --- | --- | --- |
| miRNA | 20,796 | 102.92 | 2,140,326 | 0.083475 |
| tRNA | 2,896 | 73.06 | 211,594 | 0.008252 |
| rRNA |  |  |  |  |
| rRNA | 926 | 303.27 | 280,829 | 0.010953 |
| 18S | 117 | 837 | 97,929 | 0.003819 |
| 28S | 344 | 419.55 | 144,324 | 0.005629 |
| 5.8S | 48 | 133.58 | 6,412 | 0.00025 |
| 5S | 417 | 77.13 | 32,164 | 0.001254 |
| snRNA |  |  |  |  |
| snRNA | 2,384 | 110.84 | 264,250 | 0.010306 |
| CD-box | 538 | 88.41 | 47,566 | 0.001855 |
| HACA-box | 345 | 139.62 | 48,169 | 0.001879 |
| splicing | 1,441 | 110.17 | 158,753 | 0.006192 |

Table S 10 summary statistic of functional annotated protein-coding genes

| Type | Number | Percent(%) |
| --- | --- | --- |
| Total Predicted Genes | 21,387 | - |
| Swissprot | 18,700 | 87.4 |
| Nr | 19,306 | 90.3 |
| KEGG | 16,620 | 77.7 |
| InterPro | 21,140 | 98.8 |
| GO | 19,623 | 91.8 |
| Pfam | 16,415 | 76.8 |
| Total annotated | 21,193 | 99.1 |
| Unannotated | 194 | 0.9 |
