# Supplementary material for: Genomic-transcriptomic analysis identifies the Syrian hamster as a superior animal model for human diseases
Source: BMC Genomics. 2025 Mar 24;26:286. doi: 10.1186/s12864-025-11393-4 (PMC11931762; doi:10.1186/s12864-025-11393-4)

# Supplementary Figures

Figure S1: Interchromosomal Hi-C contact map of Syrian hamster genome.

The hamster genome contig contact matrix was built using Hi-C data with Lachesis (version-201701), juicerbox(version-.11.08) and 3D-DNA(180114). The intensity of each pixel represents the number of Hi-C links of 500kb resolution in the chromosomes. The color reflects the intensity of each contact, with darker red pixels denoting higher contact probabilities. The blocks represent the contacts between one location and another. Most interactions were observed within the chromosomes.


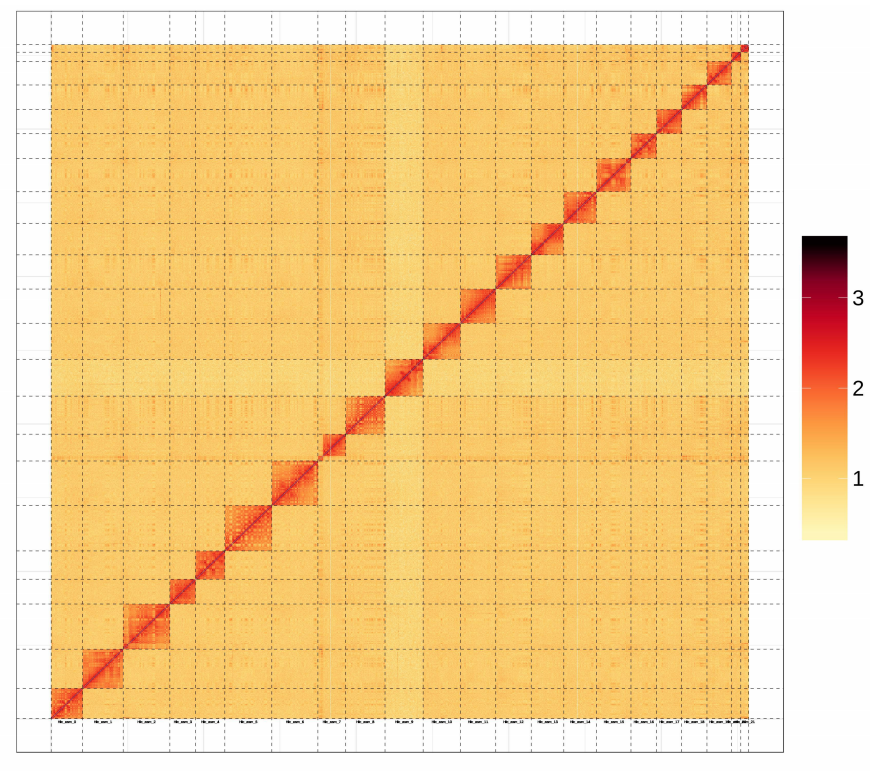


Figure S2: Heat map of genes expressed in the 15 tissues of the Syrian hamster.

The genes annotated from the 15 tissues were mapped to the assembled transcriptome of the Syrian hamster and the heatmap shows the expression level of each gene from those tissues; red color refers to up-regulated/high expression while blue color refers to down-regulated/low expression.
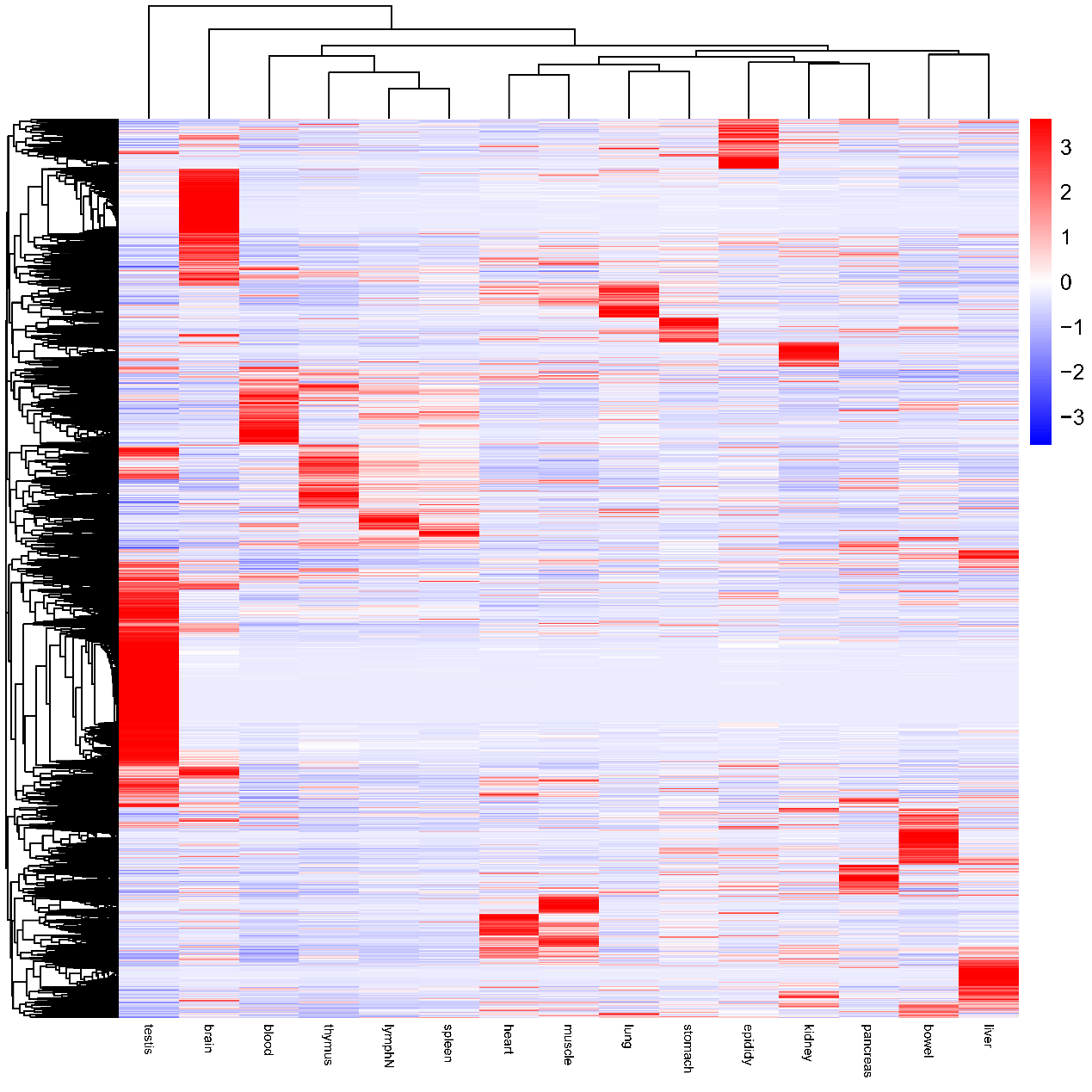


Figure S3: Expression of ACE2 in human, Syrian hamster, mouse, rat tissues and organs.

**a**. Homology alignment of ACE protein in human, mouse, rat, and Syrian hamster. **b**. The protein expression profile of ACE2 in human tissues and organs from the protein atlas database. **c.** Comparison of key binding sites of ACE2 from different species. **d-f.** Relative expression (protein and mRNA) of ACE2 in tissues and organs in Syrian hamster (**d**), mouse (**e**) and rat (**f**).
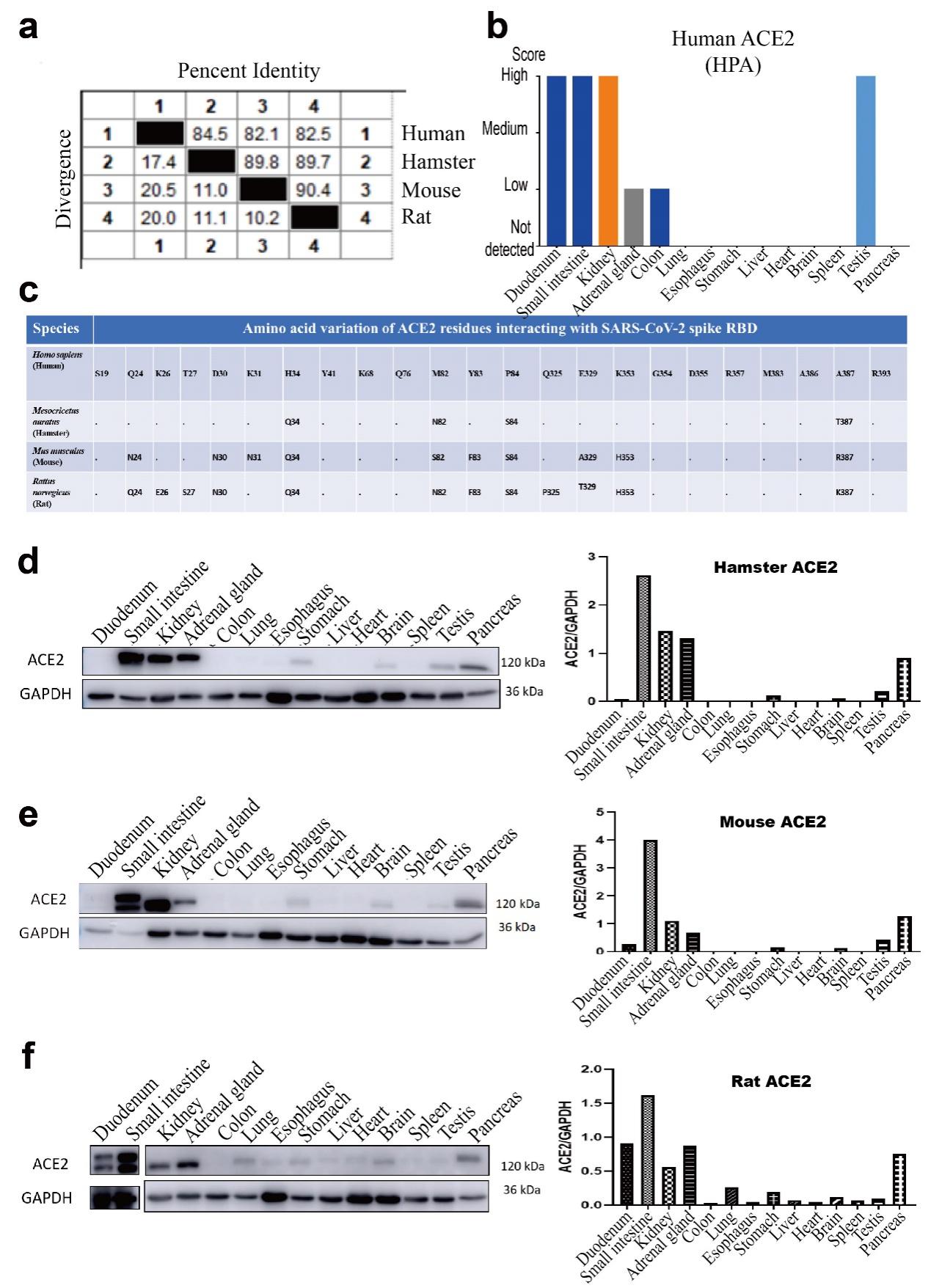


Figure S4: Characterization of Syrian hamster ACE2 receptor.

**a**. Potential binding affinity between different ACE2 variants and SARS-CoV2 spike RBD protein was analyzed with published ACE2 deep mutagenesis data. **b**. The protein expression of ACE2 in HEK-293 cells transfected with plasmids containing different ACE2 cDNA (human, mouse, rat or hamster) and different Syrian hamster-derived cell lines.


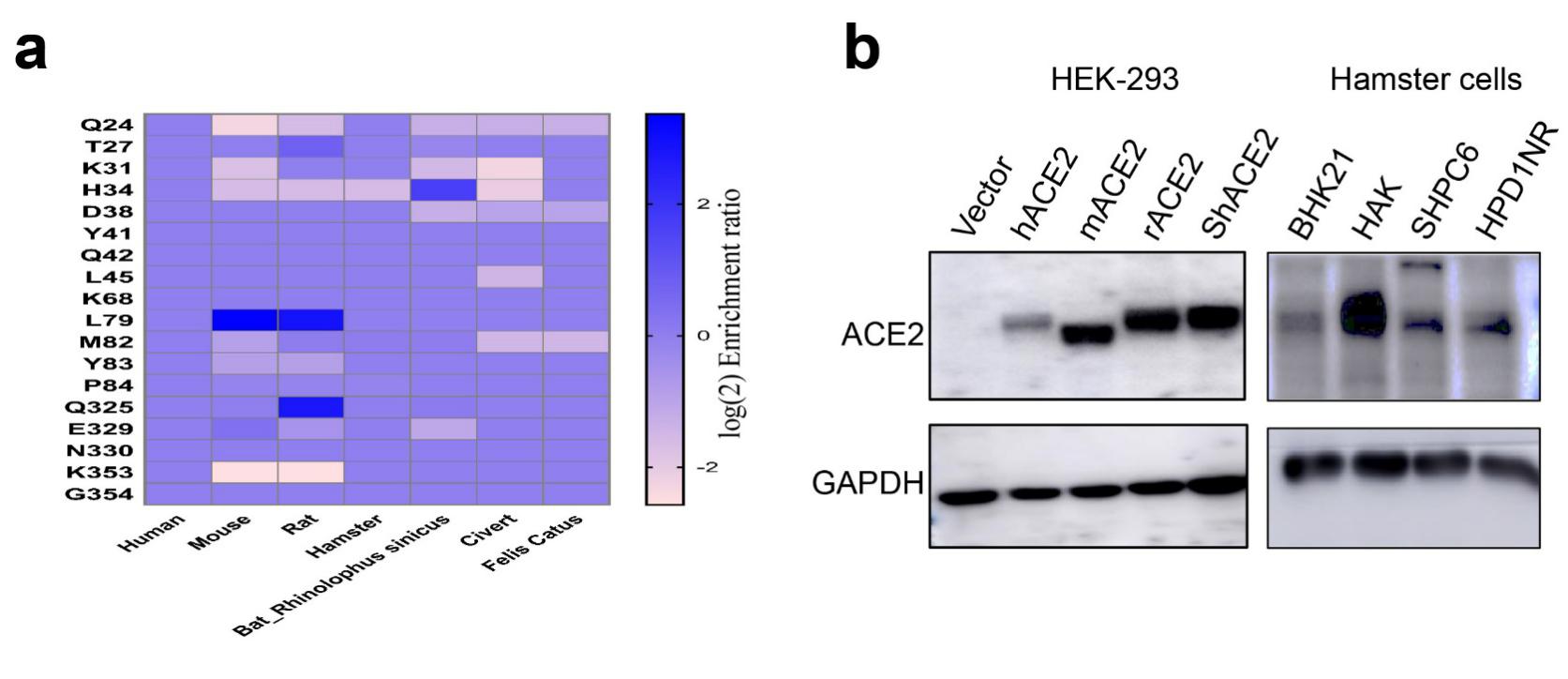


Figure S5: Characterization of Furin and MPRSS2 genes derived from different species.

**a**, Homology Alignment of Furin protease in human, mouse, rat, and Syrian hamster. **b**, Homology Alignment of TMPRSS2 protein in human, mouse, rat, and Syrian hamster. **c**, The functional domain of TMPRSS2 protease based on the public protein database. **d**, HEK-293T cells were transiently co-transfected with the plasmid expressing SARS-CoV2 spike protein and a plasmid expressing TMPRSS2 of either human, or mouse or hamster, 48 hours after transfection, total protein was harvested and detected by Western Blot using antibodies against Flag tag or S protein.
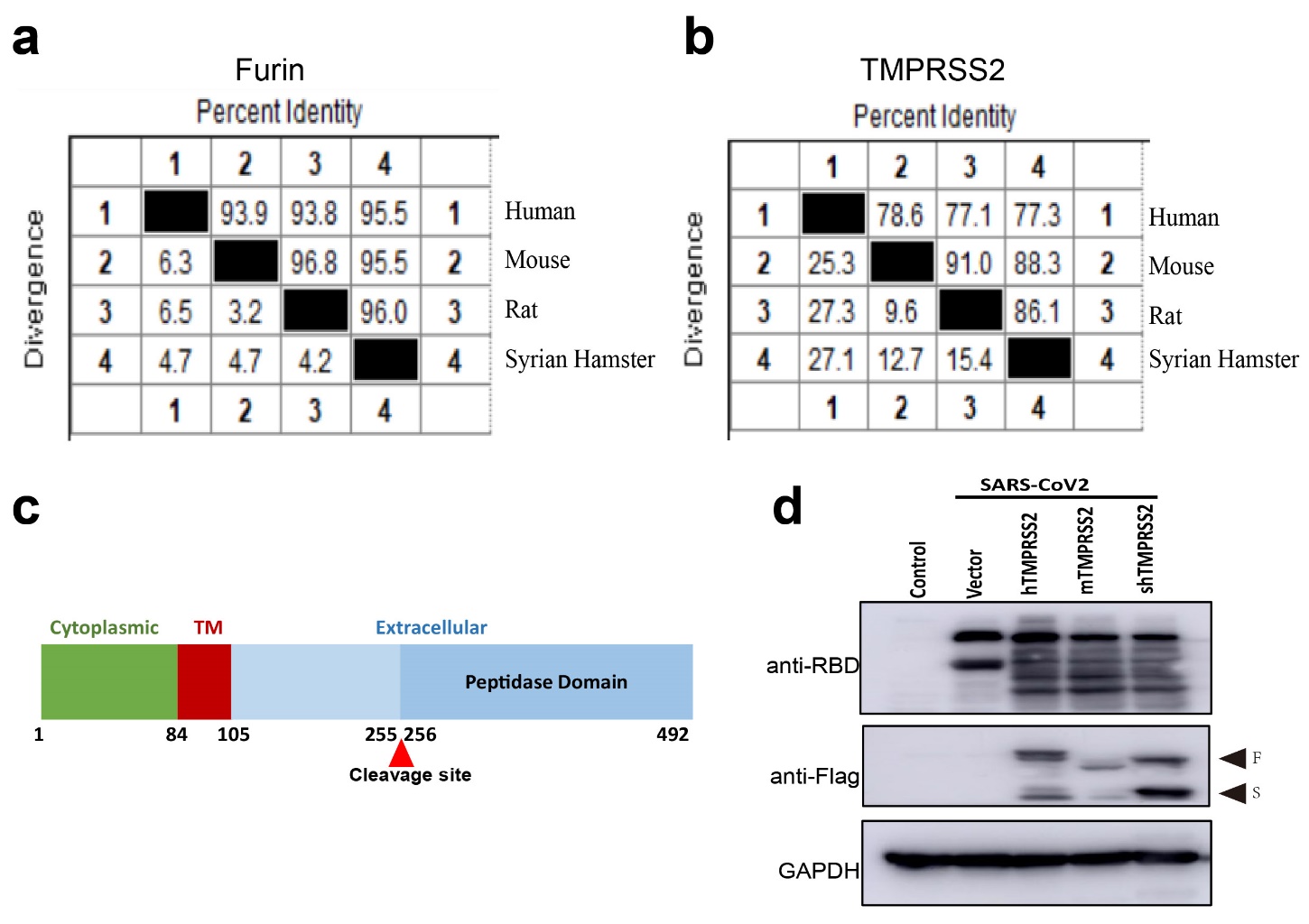

Supplement: Supplementary file 2 — Supplementary Material 2. [file 12864_2025_11393_MOESM2_ESM.docx]
